# Immune Modulation by *Plasmodium yoelii* : Insights From Lethal and Non‐Lethal Strains

**DOI:** 10.1111/pim.70045

**Published:** 2025-12-29

**Authors:** Sharoen Yu Ming Lim

**Affiliations:** ^1^ Department of Basic Medical Sciences, Faculty of Medicine and Health Sciences Universiti Malaysia Sarawak Kota Samarahan Malaysia

**Keywords:** 17XL, 17XNL, mouse model, murine malaria, *plasmodium yoelii*

## Abstract

Malaria pathogenesis is driven by intricate host–parasite interactions that determine immune balance and clinical outcome. The *Plasmodium yoelii* model, particularly its lethal (17XL) and non‐lethal (17XNL) strains, provides a robust framework to investigate these dynamics. This review integrates recent findings demonstrating that 17XL infections trigger excessive pro‐inflammatory cytokine release and immune exhaustion, while 17XNL infections sustain regulated Th1/Th2 responses enabling parasite control and survival. Emerging pathways involving MIF, TLR7 signalling, and immune checkpoints (PD‐1, LAG‐3) underscore the immunological divergence between strains. Evidence converges on a central concept: malaria severity reflects not parasite load but the timing and resolution of host immune responses. Future research using humanised models, single‐cell profiling, and immunomodulatory interventions will deepen our understanding of immune regulation and guide novel therapeutic and vaccine strategies against malaria.

## 1 Introduction

Malaria remains one of the most significant parasitic diseases worldwide, with *Plasmodium falciparum* and *Plasmodium vivax* responsible for the majority of human infections, while *Plasmodium ovale* and *Plasmodium malariae* contribute to a smaller burden [1, 2]. According to the World Malaria Report 2024, an estimated 263 million malaria cases occurred across 83 endemic countries—an increase of 11 million compared with 2022 [3]. Despite 18 countries being certified malaria‐free, the risk of reintroduction persists due to travel and migration from endemic regions. Advances in antimalarial therapies and vector control have reduced disease burden, yet the continued emergence of drug resistance and the absence of an effective vaccine sustain malaria as a major global health challenge [4, 5].

Understanding malaria pathogenesis and host immune responses is therefore critical for developing new interventions. Animal models have played a central role in this effort by enabling experimental investigations not feasible in humans. Among rodent malaria parasites, *Plasmodium yoelii*, *Plasmodium berghei*, and *Plasmodium chabaudi* are widely used to study host–parasite interactions, immunity, and drug resistance [6, 7, 8, 9]. In particular, *P. yoelii* offers a unique advantage because it exists in both lethal (17XL) and non‐lethal (17XNL) strains, providing a natural platform to study how differences in parasite virulence shape host immunity and disease outcomes [10].

These models offer practical and ethical advantages: they can be maintained in laboratory mice, allow reproducible tracking of infection dynamics, permit invasive tissue sampling, and support genetic manipulation of both parasite and host [11]. For example, progress in understanding *P. vivax* biology has been limited by the inability to establish long‐term in vitro cultures due to its preference for CD71^+^ immature red blood cells, which are scarce in peripheral circulation. Humanised mouse models that generate CD71^+^ human RBCs from transplanted HSPCs have recently provided a valuable platform for sustaining *P. vivax* infection and advancing research [12].

Each rodent malaria parasite has distinct features that make it valuable for different research questions. *P. berghei* is commonly used for cerebral malaria and transgenic studies [13]; *P. chabaudi* is a preferred model for studying chronic infection and recrudescence [14]; and *P. yoelii* is particularly useful for investigating blood‐stage immunity and vaccine responses due to the availability of both lethal and non‐lethal strains [7, 15].

This dual‐strain system provides a unique comparative platform for dissecting how parasite virulence shapes host immunity and disease outcome. Infections with the lethal 17XL strain are characterised by rapidly rising parasitemia, severe pathology, and early mortality due to uncontrolled parasite growth and dysregulated immune responses [16]. In contrast, infection with the non‐lethal 17XNL strain is typically self‐limiting, with mice developing protective immunity that can prevent reinfection [17]. This stark contrast between closely related strains offers a powerful tool to dissect how parasite virulence factors and host immune mechanisms interact to determine disease outcomes.

The ability to directly compare lethal and non‐lethal infections within the same host species makes *P. yoelii* a powerful model for unravelling the interplay between protective immunity, immune regulation, and immunopathology in malaria. The aim of this review is to summarise current knowledge on how *P. yoelii* modulates host immune responses and to highlight the contrasting outcomes observed in lethal and non‐lethal infections. By examining cytokine regulation, innate and adaptive immune mechanisms, and host–parasite interactions, this review seeks to clarify what *P. yoelii* teaches us about malaria immunopathogenesis and how insights from this model can inform the understanding of human disease.

## 2 Virulence Differences Between Strains

One of the most valuable features of *P. yoelii* as a model system is the natural variation in virulence among its strains, particularly the contrast between the lethal 17XL and non‐lethal 17XNL lines. This intrinsic variability provides a unique opportunity to study how parasite factors and host immune responses interact to determine disease outcomes. The 17XL strain is highly virulent and typically causes rapid disease progression in mice. Infections are characterised by explosive parasite replication, with parasitemia often exceeding 80%–90% within a week [18]. Such overwhelming parasitemia is associated with severe anaemia, immune suppression, and early host mortality [19, 20]. Importantly, studies show that the immune system fails to mount an effective protective response in 17XL infections. For instance, antigen‐specific T cell responses are blunted, and excessive pro‐inflammatory cytokine release contributes to immunopathology rather than protection [21]. This strain therefore serves as a useful model for fulminant malaria, where the host is unable to balance parasite clearance with immune regulation.

**FIGURE 1:**
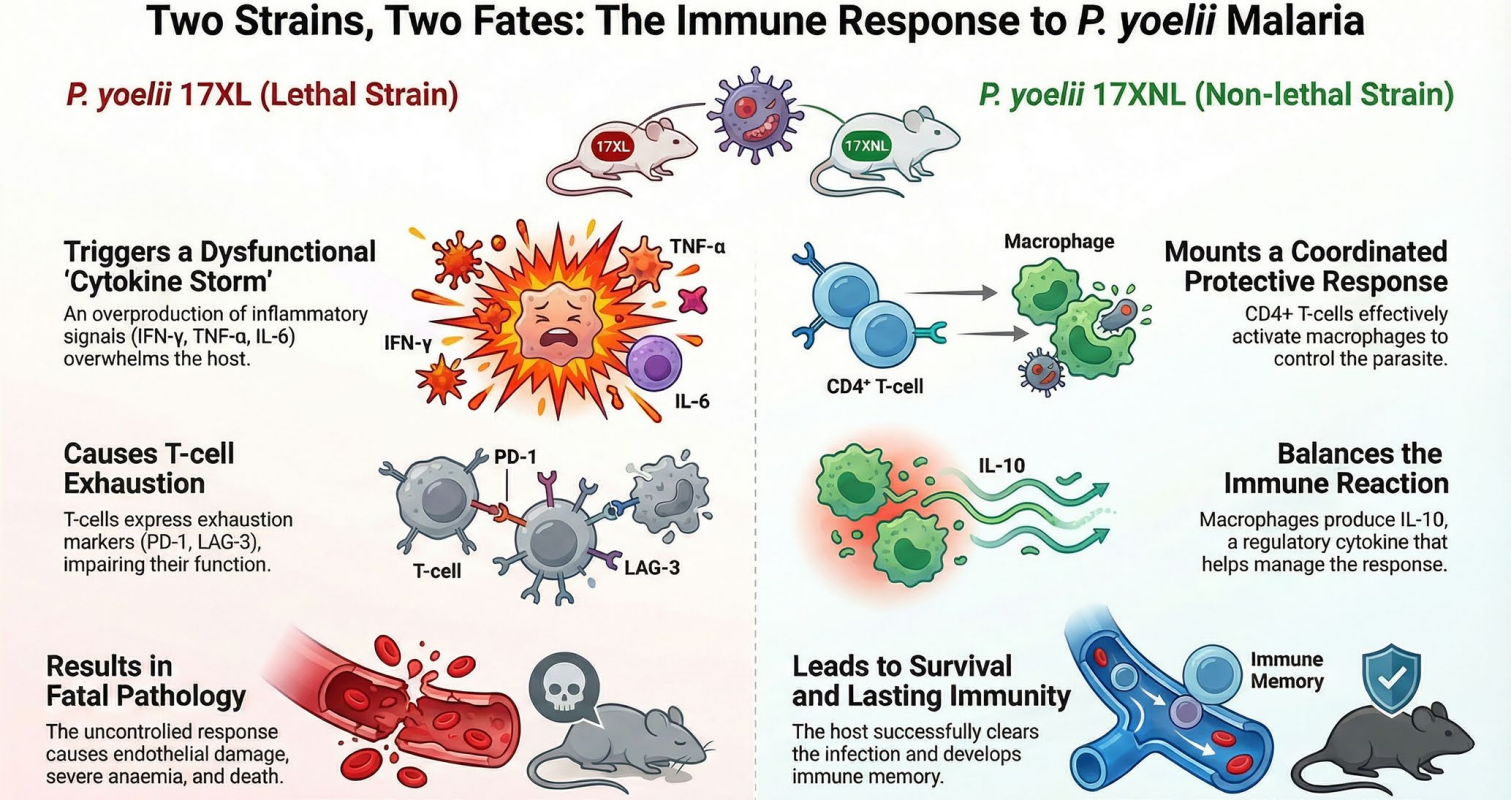
Comparative immune responses in lethal (*P. yoelii* 17XL) and non‐lethal (*P. yoelii* 17XNL) infections.

In contrast, the 17XNL strain produces a self‐limiting infection with markedly slower parasite growth [22]. Parasitemia usually peaks at moderate levels before gradually resolving, and infected mice frequently survive and acquire protective immunity [23]. This protective outcome reflects a more coordinated immune response, where both innate and adaptive arms contribute to parasite control. CD4^+^ T cell activation, B cell‐mediated antibody production, and effective regulation of pro‐inflammatory cytokines all play central roles in containing infection [24]. Importantly, recovery from 17XNL infection often leads to long‐term immunity, with the host able to resist reinfection or control subsequent challenges more efficiently [7]. Comparisons between 17XL and 17XNL infections highlight critical determinants of host–parasite interactions. In the lethal strain, immune dysregulation favours uncontrolled parasite growth and pathology, whereas in the non‐lethal strain, immune mechanisms achieve a balance between parasite clearance and host survival as shown in Figure 1. These differences emphasise that disease outcome in malaria is not solely a function of parasite replication rate, but rather the product of a dynamic interplay between parasite virulence factors and host immune responses.

By exploiting these contrasting infection phenotypes, *P. yoelii* provides an experimental platform to dissect how timing, magnitude, and regulation of immune responses influence malaria pathogenesis. This dual‐strain system allows researchers to address fundamental questions: Why does immunity succeed in one context but fail in another? Which immune pathways determine survival versus mortality? And how do parasite‐derived signals modulate these outcomes? Insights gained from these comparisons not only deepen our understanding of malaria immunobiology but also inform strategies for vaccine design and immunomodulatory therapies.

## 3 Immune Mechanisms in *P. yoelii* Infection

The outcome of *P. yoelii* infection depends largely on the nature and timing of host immune responses. Studies using lethal and non‐lethal strains have revealed distinct patterns of innate and adaptive immunity, as well as differences in cytokine regulation, which together determine whether infection resolves with protective immunity or progresses to fatal disease.

### 3.1 Innate Immunity

Innate immune responses provide the first line of defence during *P. yoelii* infection [25]. Macrophages play a dual role: they phagocytose infected erythrocytes and present antigens to T cells, but their activity is often suppressed during lethal 17XL infections, contributing to uncontrolled parasite growth. There is no consensus on the role of macrophage migration inhibitory factor (MIF) in severe malaria as some studies reported *Plasmodium* infection induces high levels of MIF in humans or mice [26] while other studies showed low MIF production increases severity of malaria infection [27]. This discrepancy could be tackled by extending the study on different *Plasmodium* strains to check the role of MIF differences across timing and magnitude of pro‐inflammatory cytokines production promoted by MIF. MIF was shown to exacerbate immunopathology in *P. yoelii* 17XL infection as knockout mice displayed reduced parasitemia, delayed mortality, and less severe anaemia and cachexia [28]. This protective effect was associated with a balanced Th1/Th2 response, characterised by higher IL‐10, IL‐12, IL‐4, and IL‐17, and lower IFN‐γ, TNF‐α, and nitric oxide levels [28]. In non‐lethal 17XNL infections, macrophages exhibit stronger activation, aiding in parasite clearance and stimulating downstream adaptive responses [29].

Dendritic cells (DCs) are crucial for priming T cell responses, but their function can be impaired in lethal infections. Evidence suggests that 17XL parasites induce DC dysfunction [30], limiting antigen presentation and blunting T cell activation. In contrast, DCs remain more functional in 17XNL infections, leading to robust activation of CD4^+^ T cells and effective adaptive immunity [31].

Natural killer (NK) cells contribute to early control of parasitemia by producing IFN‐γ and killing infected cells. However, their role differs between strains: in 17XNL infections, NK cell activity peaks early, supporting parasite control, whereas in 17XL infections NK responses are weaker and delayed, correlating with poor disease outcomes [32].

### 3.2 Adaptive Immunity

The adaptive immune system is central to determining infection outcome. CD4^+^ T cells are essential for orchestrating protective immunity [33]. In non‐lethal 17XNL infections, CD4^+^ T cells promote B cell maturation and antibody production, while also secreting cytokines that support macrophage activation. Conversely, in 17XL infections, CD4^+^ T cell responses are often suppressed or dysregulated, impairing both antibody‐mediated and cellular defences [34].

CD8^+^ T cells play a more limited role in blood‐stage infections but can contribute to parasite clearance through cytotoxic mechanisms and cytokine production [35]. Their effectiveness, however, appears reduced in lethal infections, likely due to impaired antigen presentation [36].

Antibody responses are critical in resolving non‐lethal infections. Mice infected with 17XNL generate strong IgG responses that target blood‐stage antigens [37], leading to parasite clearance and long‐lasting protection. Passive transfer of immune serum from 17XNL‐infected mice can protect naïve mice [38, 39], underscoring the importance of humoral immunity. In lethal infections, antibody responses are either delayed or insufficient to counter rapid parasite growth.

### 3.3 Cytokine Regulation

Cytokines play a decisive role in shaping infection outcomes. Transforming growth factor‐β (TGF‐β) is a particularly important regulator. In non‐lethal infections, timely TGF‐β production helps prevent excessive inflammation while allowing parasite clearance. However, in lethal 17XL infections, dysregulated or premature TGF‐β production can suppress protective responses and exacerbate disease [40, 41].

Pro‐inflammatory cytokines such as IFN‐γ and TNF‐α are essential for early parasite control. In 17XNL infections, their production is tightly regulated, resulting in effective clearance without excessive pathology [42]. In lethal infections, however, uncontrolled production can lead to immunopathology and tissue damage.

Regulatory cytokines such as IL‐10 are required to limit immune‐mediated damage. In non‐lethal infections, IL‐10 balances pro‐inflammatory responses, promoting host survival. In contrast, lethal infections are often marked by inadequate or poorly timed IL‐10 responses, contributing to unchecked inflammation and mortality [43].

Recent studies have continued to refine our understanding of how lethal and non‐lethal *P. yoelii* strains modulate host immunity. Table 1 summarises key findings from recent research (2021–2025), highlighting differences in innate and adaptive immune responses, cytokine regulation, and outcomes between 17XL and 17XNL infections.

**TABLE 1:** Recent research comparing immune responses in lethal (17XL) and non‐lethal (17XNL) *Plasmodium yoelii* infections.

Strain(s)	Immune component/parameter measured	Findings in 17XL (lethal)	Findings in 17XNL (non‐lethal)	Implications	References
17XL (lethal) and 17XNL (non‐lethal)	Th1, Th2, Tfh responses; PD‐1 expression; antibody levels; parasitemia and survival	Early peak of Th1 (Day 3) with high IFN‐γ but rapid decline; higher PD‐1 expression in adults at late stage; high parasitemia and 100% mortality	Stronger and sustained Th1 (Day 5) and Th2/Tfh responses in adults; higher IL‐4 and antibody (IgG, IgG1, IgG2a, MSP‐1–specific) levels; lower PD‐1 expression and reduced parasitemia; higher survival (96%)	Age‐related immune maturation enhances balanced Th1/Th2 and humoral responses in adults, promoting effective parasite control and survival, whereas immature immunity and higher PD‐1‐mediated exhaustion in young mice lead to severe infection and mortality	[44]
17XL and 17XNL	PD‐1/PD‐L1 and LAG‐3 inhibitory pathways; CD4^+^ and CD8^+^ T cell cytokine responses; GC B cells, TFH/TFR cells, and antibody production	LAG‐3 and PD‐L1 co‐blockade synergistically enhanced parasite clearance; blockade rescued CD4^+^ T cell cytokine production (IL‐2, TNF, IFN‐γ) but not CD8^+^ T cell function; effect independent of antibody responses; LAG‐3 expressed mainly on parasite‐specific CD4^+^ T cells (Tbet^+^, TFH, TFR)	PD‐1^−^/^−^ and PD‐L1^−^/^−^ mice showed similar infection kinetics to WT, indicating compensatory mechanisms; LAG‐3 blockade in PD‐L1^−^/^−^ mice reduced parasitemia without enhancing GC B or TFH responses; serum transfer showed no increase in protective antibodies	LAG‐3 is a dominant checkpoint regulator of CD4^+^ T cell effector function in malaria; its blockade enhances parasite clearance independent of humoral immunity, suggesting potential for targeted immunotherapy to restore T cell function and accelerate parasite elimination	[45]
17XL	PD‐1 expression and regulation in CD4^+^ T cells	Marked upregulation of PD‐1 on splenic CD4^+^ T cells with increased NFATc1 and HIF‐1α activation; PD‐1^+^ CD4^+^ T cells highly activated, secreting IFN‐γ, IL‐10, IL‐2, and IL‐4; associated with splenomegaly and severe immune modulation	Lower PD‐1 expression, minimal NFATc1 and HIF‐1α activity; balanced cytokine profile and normal spleen morphology	Severe (*17XL*) infection induces hypoxia‐driven HIF‐1α → NFATc1 → PD‐1 signalling in CD4^+^ T cells, leading to immune activation and possible T‐cell exhaustion, while milder (*17XNL*) infection maintains controlled immune responses	[46]
17XL	MIF, cytokines (IL‐12, IL‐10, IL‐17, IFN‐γ, TNF‐α, IL‐4), NO, macrophage and splenocyte function	High MIF, elevated TNF‐α and IFN‐γ, severe anaemia and splenomegaly, early death (day 11)	MIF^−^/^−^ mice: ↑IL‐12, IL‐17, IL‐10, IL‐4, ↓IFN‐γ, less splenomegaly and anaemia, delayed death (Day 21)	MIF promotes immunopathology; its absence enhances immune balance and survival	[28]
17XNL	Mast cell chymase (Mcpt4) deficiency—effects on parasitemia, intestinal barrier, cytokines, and immune regulation	Not assessed	Mcpt4^−^/^−^ mice showed ↓ parasitemia, ↑ intestinal permeability, early ↑ ileal mast cells and IgE, altered E‐cadherin/ZO‐1 junctions, ↓ neutrophil MPO activity, early Type‐1 cytokine bias (↑IL‐12p40, TNF‐α) vs. Type‐2 bias (↑IL‐10, IL‐4) in WT; despite reduced parasitemia, ↑ transmission to *A. stephensi*	Mcpt4 suppresses early Type‐1 immune response and controls neutrophil activation; its absence enhances host resistance (↓parasitemia) but increases intestinal permeability and mosquito transmission potential	[47]
*P. berghei*, *P. chabaudi*, *P. yoelii* 17XL and 17XNL	Parasitaemia, survival, ICOS expression on CD4^+^ T cells, CD4^+^ICOS^+^Foxp3^+^ regulatory T cells, cytokines (IFN‐γ, IL‐10, IL‐12)	Rapid parasitaemia rise leading to death by days 9–14 p.i.; persistent increase in CD4^+^ICOS^+^ T cells and high frequency (~50%) of CD4^+^ICOS^+^Foxp3^+^ Tregs; elevated IFN‐γ and IL‐10 with suppressed IL‐12; strong immunosuppressive profile associated with high parasite load and hemozoin accumulation	Gradual parasitaemia increase followed by clearance by day 13 p.i. with 100% survival; transient ICOS^+^ T cell activation with low Foxp3^+^ Treg frequency (~4%); moderate IFN‐γ and low IL‐10 but sustained IL‐12 production supporting parasite clearance	Lethal Plasmodium infections induce prolonged T cell activation and regulatory dominance (ICOS^+^Foxp3^+^, IL‐10), leading to immune suppression and poor survival, whereas non‐lethal infections favour a balanced pro‐inflammatory (IL‐12‐driven) response enabling parasite control and host recovery	[48]
17XL and 17XNL	Parasitaemia, survival, spleen histopathology, hemozoin accumulation, B220^+^ B cells, IL‐10^+^ regulatory B cells (Bregs), adoptive Breg transfer effects, cytokine responses (IFN‐γ, IL‐10), PD‐L1 expression	Rapid parasitaemia increase; mice die by day 13–15 p.i.; extensive spleen disruption and persistent hemozoin accumulation; continuous expansion of Bregs and high IL‐10 production; adoptive Breg transfer reduces parasitaemia and IFN‐γ, increases IL‐10; PD‐L1 expression downregulated after Breg transfer	Slower parasitaemia growth with full recovery; transient hemozoin accumulation resolving with parasite clearance; early rise but later decline in Bregs and IL‐10; balanced cytokine profile limiting pathology	Lethal infection elicits sustained inflammatory and immunosuppressive responses with uncontrolled parasite growth, while non‐lethal infection induces transient immune activation and resolution. Bregs (IL‐10^+^) modulate immunity by suppressing inflammation and enhancing survival, highlighting their potential therapeutic role in severe malaria	[49]
17X	Role of transcription factor Bhlhe40 in regulating CD4^+^ T‐cell cytokine responses (IL‐10, IFN‐γ), proliferation, and parasite control	Not assessed	Bhlhe40^−^/^−^ mice showed higher parasitemia and delayed clearance, due to increased IL‐10 production by CD4^+^ T cells and reduced IFN‐γ production in the liver. Bhlhe40 deficiency did not affect CD4^+^ T‐cell proliferation, Treg, or humoral responses. Blockade of IL‐10R restored parasite control	Bhlhe40 expression in CD4^+^ T cells is essential for efficient control of non‐lethal P. yoelii infection by suppressing IL‐10 and sustaining IFN‐γ‐mediated protective immunity	[50]
17XL	PySRA (PYYM_1014900) recombinant fragments (F1: aa24–298, F2: aa299–548, F3: aa705–852); immune protection and macrophage interaction	Only the PySRA‐F2 fragment conferred strong protection: mice immunised with F2 showed low parasitemia, 66.7% survival, and intact spleen structure, while others reached ~80% parasitemia and died by day 13. PySRA‐F2 bound to CD68 on macrophages, activated NF‐κB and MAPK pathways, induced pro‐inflammatory cytokines (IL‐1β, TNF‐α, IL‐6) and macrophage apoptosis, but also regulated inflammation to aid survival. Attempts to knock out PySRA or PySRA‐F2 failed, indicating essentiality.	Not assessed	PySRA‐F2 is an essential exported protein that interacts with macrophages via CD68, modulating inflammatory and apoptotic pathways to balance host immunity and parasite survival—highlighting its potential as a protective antigen and vaccine target in lethal malaria	[51]
Yoelii NSM	TLR7–Tfh cell axis regulating humoral immunity (Tfh cell differentiation, cytokines, B cell help, antibody production, STAT3/Ikzf2 signalling)	Not assessed	Infection caused transient accumulation of splenic Tfh cells (CXCR5^+^PD‐1^+^) peaking at Day 8, correlating with parasitemia. TLR7 highly expressed in Tfh cells; activation by R848 or iRBC lysate enhanced Tfh differentiation, CD69, IL‐21, IFN‐γ, and antibody production. TLR7^−^/^−^ mice showed ↑ parasitemia, splenomegaly, ↓ Tfh %, ↓ CD40L, IL‐4, IL‐21, IL‐10, and ↓ IgG/IgM/IgE. Mechanistically, TLR7 regulated Tfh survival via MyD88–STAT3/Ikzf2 pathway; R848 treatment restored Tfh %, plasma cells, STAT3/Ikzf2, and improved parasite control	TLR7 promotes protective humoral immunity during non‐lethal P. yoelii infection by enhancing Tfh differentiation, survival, and B‐cell help via the STAT3/Ikzf2 pathway, whereas TLR7 deficiency impairs antibody responses and increases parasitemia.	[52]
17XL and 17XNL	Platelet IDO1 mRNA expression, TRP–KYN metabolism, IFN‐γ regulation, platelet‐specific IDO1 knockout (psIdo1^−^/^−^) effects	Not specifically reported; lethal infection likely causes dysregulated TRP–KYN metabolism and severe thrombocytopenia	PyNL infection increased platelet IDO1 expression (IFN‐γ‐dependent), elevated plasma KYN and KTR, reduced serotonin and 5‐HIAA; psIdo1^−^/^−^ mice showed higher parasitemia, weight loss, thrombosis, and mortality	Platelet IDO1 shifts TRP metabolism towards the KYN pathway, limiting thrombosis and supporting infection tolerance; loss of IDO1 impairs metabolic regulation and worsens malaria outcomes	[53]
17XNL	CD4^+^ T cells/T follicular helper (Tfh) cell differentiation and signalling (RACK1–STAT3 pathway)	Not assessed	RACK1 is essential for CD4^+^ T cell activation, proliferation, and Tfh differentiation via stabilisation of STAT3 and induction of Bcl‐6. Loss of RACK1 impairs STAT3 phosphorylation, germinal centre formation, and parasite‐specific IgG production	RACK1 acts as a key post‐translational regulator of STAT3, promoting effective Tfh differentiation and humoral immunity during non‐lethal P. yoelii infection. Targeting RACK1–STAT3 axis may enhance vaccine‐induced or natural immunity to malaria	[54]
17XL and 17XNL	Anaemia, hyperlactatemia, tissue hypoxia (HIF‐1α, Glut1, Ldha, Mct4), liver/kidney injury, and lactate metabolism	Severe anaemia with peak parasitemia (~77%) and markedly high lactate (≈19 mmol/L); extensive tissue hypoxia in liver, kidney, and gut; HIF‐1α activation and glycolytic upregulation (↑Glut1, ↑Mct4, ↑lactate production); elevated ALT/AST indicating hepatic injury; hemosiderin deposits in kidney; combined artesunate + blood transfusion restored haematocrit and reduced lactate more effectively than artesunate alone	Moderate anaemia and lactate rise (≈12 mmol/L, 24% parasitemia); mild hepatic enzyme elevation and renal hemosiderin accumulation; spontaneous recovery after parasitemia decline	Anaemia is a major driver of hyperlactatemia, but in lethal P. yoelii 17XL infection, additional hypoxia‐driven glycolytic and organ‐injury mechanisms exacerbate lactate accumulation. Combined anti‐malarial and transfusion therapy enhances recovery. TFF3 correlates with lactate and malaria severity, linking gut injury to systemic metabolic dysfunction in severe malaria	[19]
17XNL and BCG co‐infection models	Mycobacterial Growth Inhibition Assay (MGIA), parasitaemia, CD4^+^ T‐cell cytokines (IFN‐γ, TNF‐α), memory subsets (Tcm, Teff)	Not applicable	BCG vaccination remained protective despite 17XNL infection; parasitaemia peaked ~20%–28% but resolved by Day 21. 17XNL infection after BCG did not impair ex vivo mycobacterial control, but infection during BCG vaccination reduced TNF‐α and total cytokine responses, mirroring impaired bacterial control. Cleared malaria before vaccination restored normal responses. No major effects on memory (Tcm/Teff) subsets	Mild malaria infection does not compromise BCG efficacy if vaccination occurs after infection resolution, but acute infection during vaccination blunts Th1 cytokine responses and reduces vaccine efficacy, emphasising timing importance in malaria‐endemic TB vaccination programmes	[55]
17XL and 17XNL	miRNA expression profiles in splenocytes (day 13 post‐infection)	171 miRNAs differentially expressed vs. controls; 53 uniquely dysregulated; key upregulated miRNAs: miR‐6992–5p, miR‐704, miR‐7676–3p, miR‐1933–3p. These target immune and inflammatory pathways (MAPK, mTOR, TGF‐β), possibly linked to severe pathology	220 miRNAs differentially expressed vs. controls; 86 uniquely dysregulated; key upregulated miRNAs: miR‐875–3p, miR‐30a‐3p, miR‐499–5p, miR‐212–3p. Enriched in immune regulation and cell survival pathways (MAPK, mTOR, FoxO, Th17 differentiation)	Distinct miRNA signatures distinguish lethal from non‐lethal P. yoelii infections, reflecting strain‐specific immune regulation. These miRNAs could serve as biomarkers or therapeutic targets for malaria severity and immune modulation	[56]

## 4 Host–Pathogen Interactions: Lessons Learned

Parasite genetic differences play a central role in determining infection outcome. Variations between the 17XL and 17XNL strains influence not only parasite replication rates but also their ability to modulate host immune responses [57]. Lethal strains appear to suppress antigen presentation and T cell activation, impairing the development of protective immunity. In addition, parasite‐derived molecules can skew dendritic cells and macrophage function, creating an environment where immune evasion is favoured [58]. These findings highlight that parasite virulence is not merely a reflection of growth kinetics but also involves active strategies to subvert host immunity.

The kinetics of cytokine responses are crucial in determining whether infection resolves or progresses to fatal disease. In non‐lethal infections, early production of pro‐inflammatory cytokines such as IFN‐γ and TNF‐α supports parasite control, while timely induction of regulatory cytokines like IL‐10 and TGF‐β prevents excessive inflammation [15]. In lethal infections, however, cytokine production is often dysregulated, either delayed, excessively, or mistimed [59]. Premature TGF‐β release can suppress protective immunity, while uncontrolled IFN‐γ and TNF‐α drive immunopathology [31, 60]. Thus, it is not only the presence of cytokines but also their precise temporal coordination that determines outcomes.

**FIGURE 2:**
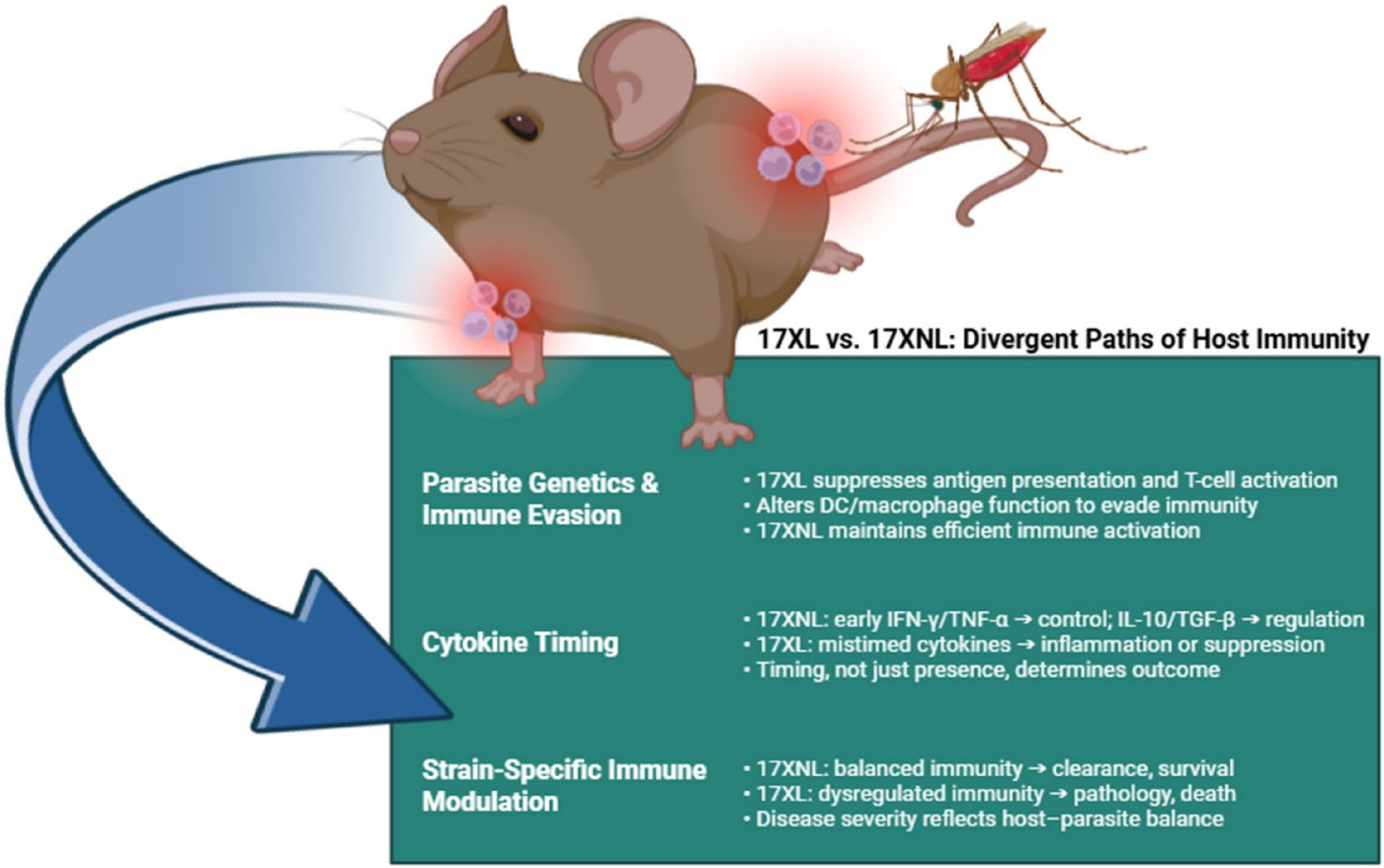
Comparative summary of host–pathogen interactions in *Plasmodium yoelii* strains.

Comparisons between 17XL and 17XNL infections illustrate how closely related parasite strains can elicit fundamentally different immune landscapes. In non‐lethal infections, the host immune system is able to mount a balanced response that clears parasites while minimising tissue damage. In contrast, lethal infections reveal how immune suppression and dysregulation contribute to uncontrolled parasitemia and mortality. This strain‐dependent modulation provides a “window” into malaria immunopathology, demonstrating that disease severity results from a delicate balance between parasite strategies of immune evasion and host mechanisms of immune regulation. Key aspects of host–pathogen interactions that distinguish lethal from non‐lethal *P. yoelii* infections are summarised in Figure 2.

The *P. yoelii* model demonstrates that malaria pathogenesis is shaped by both parasite virulence traits and host immune responses, with timing and balance being critical determinants of outcome. These lessons extend to human malaria, where variability in host genetics, immune history, and parasite diversity also influence clinical manifestations [61]. By dissecting the contrasting host–pathogen interactions in *P. yoelii*, researchers gain valuable clues about the mechanisms that govern protective immunity versus severe disease, offering pathways for vaccine and therapeutic development.

## 5 Translational Relevance to Human Malaria

### 5.1 Immunological Parallels Between *P. yoelii* and Human Malaria

Studies of *P. yoelii* have revealed several immunological processes that closely resemble those seen in human malaria. One important parallel is the occurrence of cytokine storms. Infections with lethal strains such as 17XL often trigger massive production of pro‐inflammatory cytokines like IFN‐γ and TNF‐α, leading to systemic inflammation and tissue damage. This pattern mirrors the immunopathology of severe *P. falciparum* infections, in which an uncontrolled inflammation cascade contributes significantly to complications and death [62]. Another shared feature is the role of regulatory cytokines. Both in mice and in humans, IL‐10 and TGF‐β can tip the balance between protection and pathology [43]. When tightly regulated, these cytokines protect the host from immune‐mediated damage, but their mistimed or excessive production can allow persistent parasitemia and increase disease severity [63].

Further similarity lies in the phenomenon of immune exhaustion. During chronic *P. yoelii* infection, sustained antigen exposure weakens T cells and dendritic cell function, limiting the ability of the immune system to mount effective responses. Comparable immune exhaustion has been documented in chronic *P. falciparum* infection, where antigen persistence and immune suppression hinder parasite clearance. The parasite population exhausted its variant antigen repertoire; parasites can stop expressing PfEMP1, enabling them to avoid recognition by PfEMP1‐specific antibodies and thus allowing them to persist indefinitely despite a robust antibody response [64]. Finally, the development of protective immunity in non‐lethal *P. yoelii* infections reflects the gradual acquisition of immunity in humans living in endemic regions [44]. In both cases, strong antibody responses play a central role, clearing parasites and conferring long‐term protection against reinfection.

### 5.2 Differences and Limitations of the Model

Despite these parallels, *P. yoelii* does not fully replicate the complexity of human malaria. The murine immune system differs from that of humans in the composition of T cell subsets, cytokine networks, and innate responses, which can exaggerate or diminish the role of certain pathways [65, 66, 67]. In addition, the clinical manifestations of *P. yoelii* infection diverge from those of *P. falciparum*. Rodent malaria causes severe malaria without cerebral complications termed non‐cerebral malaria (NCM) [68]. Unlike humans who remain susceptible to repeat *Plasmodium* infections, mice generate sterilising immunity to *P. yoelii* after just one exposure, so a cross‐species *Plasmodium* challenge is used to study the immune response to reinfection [69].

Another limitation is parasite biology. Unlike *P. falciparum*, which relies on extensive antigenic variation through var. genes and PfEMP1 proteins [70], *P. yoelii* lacks the extensive antigenic variation seen in *P. falciparum*, a key strategy for human malaria parasites to evade the host immune response through constant changes in their surface proteins [71, 72]. This difference means that, while *P. yoelii* uses some immune evasion tactics, its simpler strategies make it a less effective mimic of the more complex immune evasion by human malaria parasites. Laboratory conditions further constrain the model [66, 73]. Infections in genetically identical mice under controlled environments lack the variability present in human populations, where genetic background, nutritional status, and co‐infections influence disease outcomes.

### 5.3 Value as a Comparative Model

Although *P. yoelii* cannot serve as a perfect substitute for human malaria, its strength lies in its role as a comparative model that highlights universal principles of host–parasite interaction. The coexistence of lethal and non‐lethal strains provides a unique system to explore how immune regulation dictates disease outcomes. Few malaria models allow such direct comparisons, making *P. yoelii* particularly valuable for dissecting mechanisms of pathogenesis and protection. Furthermore, advances in mouse genetics make it possible to selectively delete or overexpress immune mediators, allowing precise investigation of their roles in infection. These findings frequently inform hypotheses that can later be tested in human studies. Finally, *P. yoelii* continues to serve as a pre‐clinical tool for evaluating vaccines and immunotherapies. The well‐characterised antibody responses that mediate protection in non‐lethal infections provide a robust platform for testing the efficacy of candidate interventions.

### 5.4 Integrative Perspective

The translational value of *P. yoelii* does not lie in reproducing every aspect of human malaria but rather in illuminating conserved immune mechanisms and pathophysiological processes. By offering a controlled experimental system in which infection outcomes can be compared and manipulated, the model provides insights that complement findings from *P. falciparum* studies, non‐human primates, and human clinical cohorts. Its contribution is therefore best understood as part of a broader research landscape. When used in this way, *P. yoelii* continues to play a critical role in advancing our understanding of malaria immunopathogenesis and in guiding the development of strategies for prevention and treatment.

## 6 Future Perspectives and Conclusion

The study of *P. yoelii* is poised to benefit greatly from recent advances in experimental technologies. Genome‐editing platforms such as CRISPR–Cas9 now make it possible to manipulate parasite genes with high precision, offering opportunities to identify virulence determinants and dissect pathways involved in immune evasion [74, 75]. In parallel, high‐throughput transcriptomics and proteomics provide a systems‐level view of host–parasite interactions, capturing the dynamic changes that occur during infection. Perhaps the most transformative is the application of single‐cell sequencing, which allows researchers to map the heterogeneity of immune responses at unprecedented resolution [76, 77]. Together, these tools will enable a deeper understanding of how lethal and non‐lethal strains of *P. yoelii* drive divergent immune outcomes and how host responses can be fine‐tuned towards protection rather than pathology.

Beyond its role in basic immunology, *P. yoelii* remains a valuable platform for translational research. The ability of non‐lethal infections to generate strong antibody responses and long‐term immunity makes this model particularly suitable for evaluating vaccine candidates. It allows for testing not only the induction of protective humoral and cellular immunity but also the durability and breadth of responses upon reinfection [7]. Furthermore, the model provides a controlled setting for pre‐clinical assessment of immunotherapies aimed at modulating host responses, including therapies designed to enhance regulatory pathways, restore T cell function, or rebalance cytokine production, which can be systematically studied in the *P. yoelii* system before moving into human trials.

For *P. yoelii* research to retain its relevance, findings must increasingly be placed in dialogue with human malaria studies. Comparative analyses that integrate murine data with samples from malaria patients, such as cytokine profiles, immune cell phenotypes, and genetic signatures, will be critical to identify which mechanisms are conserved and which are species‐specific. Such cross‐validation will help bridge the gap between experimental models and clinical reality, ensuring that discoveries in *P. yoelii* can inform interventions for human disease more directly.

In conclusion, *P. yoelii* offers a unique advantage in malaria research through its dual‐strain system, which naturally contrasts lethal and non‐lethal outcomes within the same parasite species. This model has revealed a central lesson that applies across malaria systems: the timing and regulation of immune responses, rather than their mere presence, often determine whether an infection is cleared or progresses to severe disease. Although limitations exist due to differences between mouse and human biology, *P. yoelii* continues to provide powerful insights into the principles of malaria immunology. With the integration of modern technologies and closer alignment to human studies, this model is well‐positioned to remain a cornerstone in the effort to understand and ultimately control malaria.

## Author Contributions

**Sharoen Yu Ming Lim:** conceptualisation, visualisation, database searched, manuscript drafting, manuscript review and editing.

## Conflicts of Interest

The author declares no conflicts of interest.
